# Photon-Counting Computed Tomography of the Paranasal Sinuses Improves Intraoperative Accuracy of Image-Guided Surgery

**DOI:** 10.3390/diagnostics15212777

**Published:** 2025-10-31

**Authors:** Benjamin Philipp Ernst, Iris Burck, Stefanie Schliwa, Sven Becker, Tobias Albrecht, Thomas J. Vogl, Jan-Erik Scholtz, Anna Levi, Andreas German Loth, Friederike Bärhold, Sebastian Strieth, Matthias F. Froelich, Alexander Hertel, Yannik Christian Layer, Daniel Kuetting, Jonas Eckrich

**Affiliations:** 1 Department of Otorhinolaryngology, University Medical Center, Goethe-University Frankfurt, Theodor-Stern-Kai 7, 60590 Frankfurt, Germany; 2 Department of Radiology and Nuclear Medicine, University Medical Center, Goethe-University Frankfurt, Theodor-Stern-Kai 7, 60590 Frankfurt, Germany; 3 Institute of Anatomy and Cell Biology, University of Bonn, Nussallee 10, 53115 Bonn, Germany; 4 Department of Otorhinolaryngology, Head and Neck Surgery, University of Tübingen Medical Center, Elfriede-Aulhorn-Straße 5, 72076 Tübingen, Germany; 5 Department of Otorhinolaryngology, University Medical Center Bonn, Venusberg-Campus 1, 53127 Bonn, Germany; 6 Department of Radiology and Nuclear Medicine, University Medical Center Mannheim, Medical Faculty Mannheim, Heidelberg University, Theodor-Kutzer-Ufer 1-3, 68167 Mannheim, Germany; 7 Department of Radiology, University Medical Center Bonn, Venusberg-Campus 1, 53127 Bonn, Germany; 8 Department of Otorhinolaryngology, University Medical Center Mainz, Langenbeckstraße 1, 55131 Mainz, Germany

**Keywords:** head and neck, chronic rhinosinusitis, photon-counting CT, image-guided surgery, accuracy

## Abstract

**Background:** Computed tomography (CT)-based image-guided surgery (IGS) is of great importance in functional endoscopic sinus surgery (FESS) and requires IGS-specific imaging protocols to ensure high intraoperative accuracy. This study aimed to compare photon-counting CT (PCCT), dual-energy dual-source CT (DECT), and spectral detector CT (SDCT) of the paranasal sinuses with respect to image quality, IGS accuracy and radiation dose. **Methods:** A formalin-fixed cadaver skull was examined using PCCT, DECT and SDCT at 100 kV tube voltage with descending tube currents (mAs). The setup of electromagnetic IGS was evaluated using a visual analog scale. Accuracy was analyzed endoscopically using defined anatomical landmarks. Diagnostic image quality as well as bone and soft tissue noise were assessed qualitatively using a 5-point Likert scale and quantitatively by determination of signal-to-noise ratio. Radiation dose was evaluated using the dose length product. **Results:** While PCCT datasets could be registered and navigated accurately down to 10 mAs (1.5 mm error at 10 mAs), both DECT and SDCT exhibited significantly increased inaccuracies below 40 mAs (4.35/5.15 mm for DECT/SDCT at 25 mAs). Using PCCT therefore enabled a 45% radiation dose reduction at the minimally required dose length product using PCCT. Quantitative and qualitative image quality were superior for PCCT compared to DECT and SDCT. **Conclusions:** PCCT provides excellent accuracy of anatomical landmarks in IGS with superior image quality of the paranasal sinuses in low-mA scans and substantially reduced radiation exposure.

## 1. Background

Functional endoscopic sinus surgery (FESS) is the gold standard for a variety of sinonasal diseases, including chronic rhinosinusitis (CRS) and tumors of the paranasal sinuses [1,2]. It also forms the basis of modern concepts in endoscopic anterior skull base surgery (ESBS). In conjunction with nasal endoscopy, computed tomography (CT) confirms the diagnosis and provides anatomical details for surgical planning and prevention of intraoperative complications [3,4,5,6,7,8,9,10]. Additionally, CT imaging is a prerequisite for the use of image-guided surgery (IGS). IGS increases the accuracy during FESS and ESBS, minimizes the risk of damage to surrounding structures, and is of great value in surgical education [11,12,13]. However, pre-existing CT studies may be inadequate for IGS use, necessitating an additional CT scan according to IGS-specific protocols, with an associated increase in radiation dose that may entail potential long-term risks [14,15,16,17].

Photon-counting CT (PCCT) technology presents several inherent advantages over energy-integrating detector (EID) systems that may enhance imaging in otolaryngology. Unlike EID systems, which rely on scintillator-based indirect conversion leading to signal loss, PCCT uses cadmium telluride (CdTe) detector elements that permit for direct energy discrimination at the individual photon level. These advantages include improved spatial resolution, increased photon sensitivity, enhanced noise reduction, and the capability for inherent spectral imaging [18]. The enhanced spatial resolution and contrast-to-noise ratio (CNR) are particularly valuable when imaging delicate anatomical structures such as ethmoid cells. Furthermore, PCCT significantly reduces dental artifacts [19,20,21]. Therefore, PCCT imaging may significantly improve surgical planning of IGS-based FESS procedures.

The objective of this study was to systematically evaluate the performance of IGS based on PCCT, dual-energy dual-source CT (DECT), and spectral detector CT (SDCT) imaging. Specifically, the accuracy and feasibility of IGS, the qualitative and quantitative image quality, and the minimum radiation dose required for clinically acceptable IGS use in FESS were assessed.

## 2. Methods

### 2.1. Study Design and Study Sample

Ethical approval was obtained from the local institutional review board (Ethics Committee of the Medical Faculty of the University of Bonn, application number 187/23-EP, 30 May 2023). All procedures performed in studies involving human participants were in accordance with the ethical standards of the institutional national research committee and with the 1964 Helsinki Declaration and its later amendments or comparable ethical standards. A formalin-fixed adult cadaveric skull of a 73-year-old male was examined using different clinically approved CT scanners.

### 2.2. Imaging Protocols

CT examinations of the paranasal sinuses of the adult cadaveric skull were performed using three different scanner platforms: a DECT scanner (SOMATOM Force; Siemens Healthineers AG, Forchheim, Germany), a photon-counting CT scanner (NAEOTOM Alpha; Siemens Healthineers AG), and an SDCT scanner (IQon Spectral CT; Philips Healthcare, Best, The Netherlands). All scans were performed with a fixed tube voltage of 100 kV and a fixed tube current ranging from 10 to 30 mAs in steps of 5 mAs and from 30 to 100 mAs in steps of 10 mAs. Automatic tube current modulation was deactivated. Tin filters were used for X-ray beam hardening for PCCT and DECT; this feature is not available for the SDCT system used in this study.

Scanner-specific parameters were as follows: pitch was 0.85 for PCCT and DECT, and 0.296 for SDCT; collimation was 120 × 0.4 mm for PCCT, 24 × 0.6 mm for DECT, and 64 × 0.625 mm for SDCT; gantry rotation times ranged from 0.5 to 1.0 s.

The examination region extended from the vertex to the mandible. A slice thickness of 1.0 mm and a reconstruction increment of 0.5 mm were used for image reconstruction. For PCCT and DECT scanners, images were reconstructed using both a standard soft-tissue kernel (e.g., Siemens Br40, Philips C) and a bone kernel (e.g., Siemens Br60/Br66, Philips YC). Radiation dose parameters, including the CT dose index-volume (CTDI_vol) and dose length product (DLP), were recorded for all scans.

### 2.3. Setup of Image-Guided Surgery and Endoscopic Evaluation of Accuracy

The setup of electromagnetic IGS (NAV1^®^ electromagnetic, Karl Storz SE & Co KG, Tuttlingen, Germany) was carried out using the surface matching technique by two experienced anterior skull base surgeons. The IGS setup process was evaluated using a visual analog scale (VAS). Accuracy was analyzed endoscopically using defined anatomical landmarks on both sides by three experienced anterior skull base surgeons. These included the axilla of the middle turbinate and the corner pocket (junction of the skull base and the lamina papyracea in the sphenoid sinus), to account for both rostral and dorsal accuracy [22]. Using a straight EM-navigated probe (Karl Storz SE & Co KG, Tuttlingen, Germany), each landmark was marked under endoscopic control and a screenshot of the IGS position was acquired, resulting in *n* = 12 measurements. Accuracy was determined by measuring the distance between the actual landmark and the IGS position in all three planes.

### 2.4. Analysis of Image Quality

All CT datasets were evaluated both subjectively and objectively by three radiologists with five to ten years of experience in head and neck imaging. CT datasets were assessed independently using a dedicated PACS viewer (Centricity RIS-i 7.0, GE Healthcare, Chicago, IL, USA). CT images were presented in a random order with preset window settings (width: 2700; center: 700 HU), which the readers could adjust individually. The radiologists were blinded to the CT scanner and the acquisition parameters.

Important landmarks representing critical anatomical structures at risk during FESS were evaluated subjectively. These landmarks are crucial for assessing surgical accuracy and include the anterior skull base (Keros classification), the lamina papyracea, orbital fat, the anterior ethmoidal artery, the bony coverage of the optic nerve, and the internal carotid artery. Overall image quality, as well as metal and motion artifacts, were also evaluated. This was performed using 5-point Likert scales as previously described [23]:

- Visibility of the anterior skull base, lamina papyracea, orbital fat, anterior ethmoidal artery and bony coverage of the optic nerve and internal carotid artery (1 = not visible; 2 = vaguely visible, but not over the entire extent; 3 = vaguely visible, over the entire extent; 4 = unambiguously visible, but not over the entire extent; 5 = unambiguously visible, over the entire extent).
- Overall image quality (1 = poor; 2 = acceptable; 3 = moderate; 4 = good; 5 = excellent).
- Metal and motion artifacts (1 = severe artifacts, image interpretation impossible; 2 = severe artifacts, strong impairment of image interpretation; 3 = severe artifacts, slight impairment of image interpretation; 4 = slight artifacts, no impairment of image interpretation; 5 = no artifacts).

To evaluate the images objectively, the signal-to-noise ratio (SNR) of selected anatomical structures was calculated. Circular regions of interest with a diameter of 5 mm were drawn in consistent locations on homogeneous bone areas including the bone of the anterior skull base and retrobulbar fat. Image noise was defined as the standard deviation within the background (air). All measurements were performed twice and averaged. The following formula was used to calculate the SNR [24]:

$$SNR=\;\frac{HU\;(retrobulbar\;fat)}{SD\;(background)}$$

SDCT scans were only evaluated subjectively due to incomparability.

To determine the extent of radiation reduction, the lowest DLP required to perform a successful IGS setup with adequate accuracy for routine clinical use was evaluated for each CT system.

### 2.5. Statistical Analysis

Data are reported as the mean values ± SD. Repeated-measures one-way ANOVA was used to compare IGS accuracy and qualitative image ratings among the three CT systems (PCCT, DECT, SDCT) at each tube current level. Upon significance, Bonferroni-corrected post hoc paired t-tests were used for pairwise comparisons. A *p*-value < 0.05 was considered statistically significant. Fleiss’ κ was used to evaluate interrater reliability (IRR). All statistical analyses were performed using Prism 10 (GraphPad Software Inc., San Diego, CA, USA). Graphical presentation was performed with Microsoft Excel (Microsoft Corp., Redmond WA, USA).

## 3. Results

### 3.1. IGS Setup

**Figure 1:**
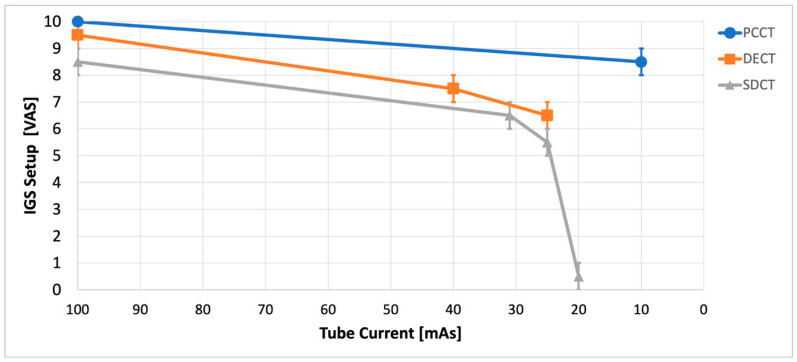
Analysis of image-guided surgery (IGS) setup using a visual analog scale (VAS) by *n* = 3 experienced skull base surgeons regarding tube currents. IGS was easily setup for high tube currents using photon-counting computed tomography (PCCT), dual-energy dual-source computed tomography (DECT) and spectral detector computed tomography (SDCT). At low currents, the ability to set up IGS deteriorates for DECT and especially for SDCT. PCCT-based IGS setup can be carried out easily up to the lowest used tube currents.

At the maximum tube current (100 mAs), the datasets of all CTs were successfully registered (VAS 10/9.5/8.5 for PCCT/DECT/SDCT, see Figure 1). PCCT-based IGS setup was successful even at the lowest tested tube current (10 mAs), corresponding to the lowest radiation exposure. In contrast, DECT- and SDCT-based IGS registration tended to become more difficult with decreasing tube currents. IGS setup was eventually impossible below 25 mAs for DECT-based IGS and below 20 mAs for SDCT-based IGS. Analysis of IRR revealed an almost perfect agreement (Fleiss‘ κ 0.82).

### 3.2. IGS Accuracy

**Figure 2:**
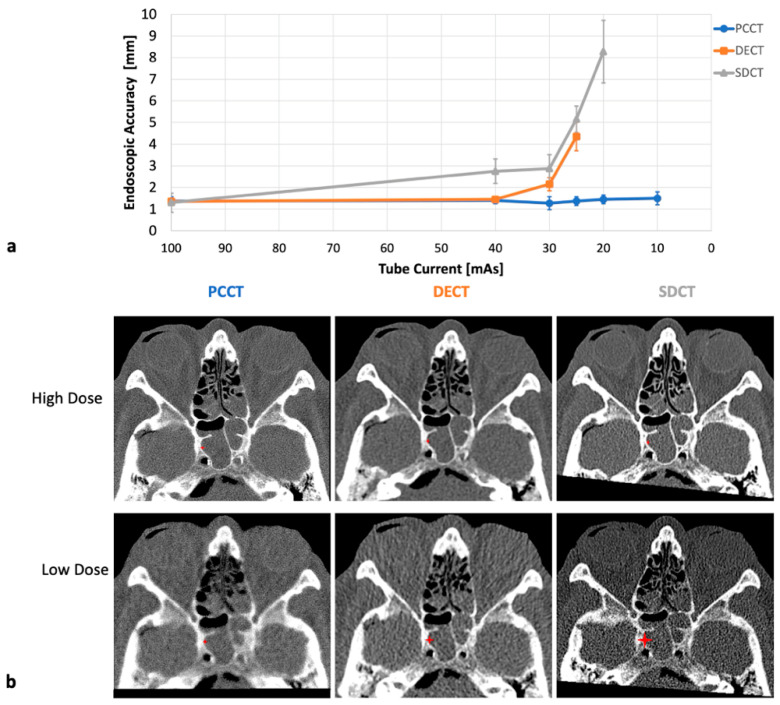
Evaluation of endoscopic accuracy of image-guided surgery by *n* = 3 experienced skull base surgeons regarding tube currents. IGS achieved very high accuracies for high tube currents using photon-counting computed tomography (PCCT, blue), dual-energy dual-source computed tomography (DECT, orange) and spectral detector computed tomography (SDCT, gray). PCCT-based IGS setup can be carried out easily up to the lowest used tube current of 10 mAs (1.5 mm). At tube currents below 40 mAs, IGS accuracy deteriorates for DECT and especially for SDCT. While IGS setup was successfully carried out down to 10 mAs for PCCT, it was not feasible below 25 mAs for DECT (4.35 mm) and below 20 mAs for SDCT (5.15 mm). Therefore, no accuracy data are available at these settings. (**a**) Quantitative IGS accuracy results as a function of tube current. (**b**) Corresponding high-dose (100 kV, 100 mAs) and low-dose sample images (100 kV, 10/25/20 mAs for PCCT/DECT/SDCT). The DECT (20 mAs) and SDCT (25 mAs) examples illustrate the lowest tube current for which IGS use was possible. Red asterisks indicate the reference anatomical landmarks and their scaled accuracies in the lateral aspect of the sphenoid sinus.

With a set tube voltage of 100 kV and a maximum tube current of 100 mAs, IGS could be used with a high accuracy using all three CTs (mean navigation error 1.35/1.375/1.3 mm for PCCT/DECT/SDCT, *p* = 0.79). While the PCCT datasets could be navigated accurately with tube currents as low as 10 mAs, both DECT and SDCT showed progressive inaccuracies below 40 mAs (see Figure 2). At 25 mAs, mean accuracies decreased to 4.35 mm for DECT and 5.15 mm for SDCT, compared to 1.5 mm for PCCT (*p* < 0.001). Consequently, these settings make the use of IGS impossible in clinical routine using DECT and SDCT imaging. Accuracy measurements were not possible for DECT-based IGS below 25 mAs and for SDCT-based IGS below 20 mAs because registration failed as described above.

### 3.3. Analysis of Image Quality and Minimally Needed Radiation Dose

**Figure 3:**
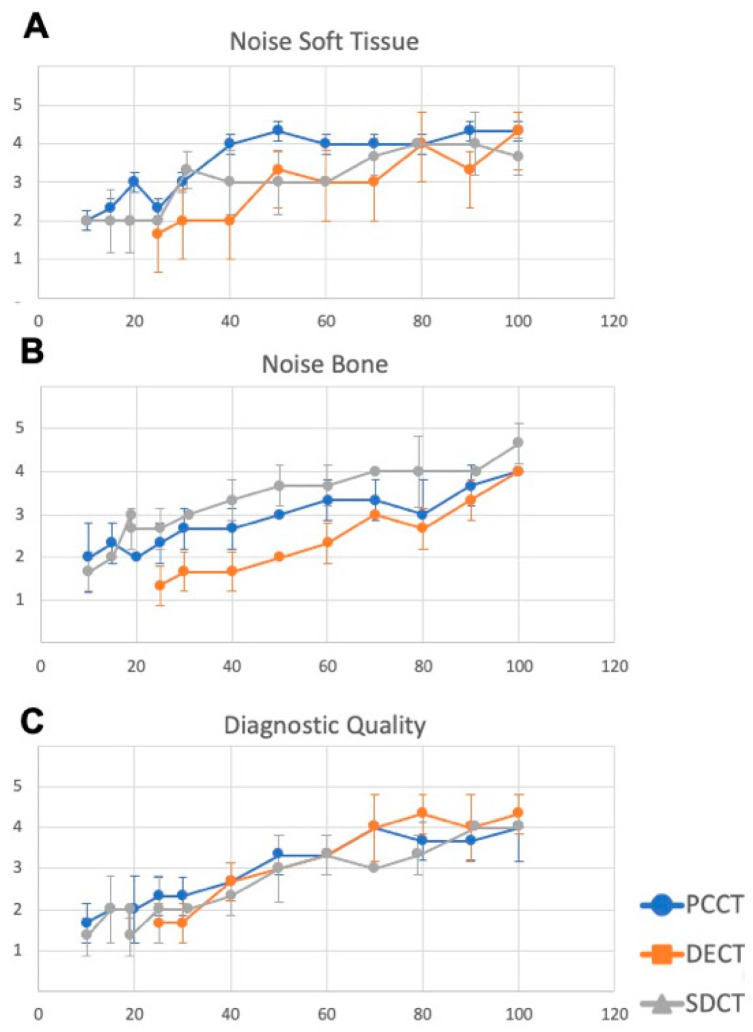
Qualitative image analysis of photon-counting computed tomography (PCCT), dual-energy dual-source computed tomography (DECT) and spectral detector computed tomography (SDCT) using a 5-point Likert scale (1 = poor, 5 = excellent). PCCT exhibits a tendency towards superior image quality in terms of soft tissue noise (**A**), SDCT for bone noise (**B**) and DECT for diagnostic quality (**C**) without reaching significance level.

Overall qualitative image quality was high for image datasets with maximum tube current for all three CT scanners in terms of soft tissue noise (4.33 vs. 4.33 vs. 3.66 for PCCT/DECT/SDCT), bone noise (4.0 vs. 4.0 vs. 4.66 for PCCT/DECT/SDCT) and diagnostic quality (4.0 vs. 4.33 vs. 4.0 for PCCT/DECT/SDCT), respectively. While all these parameters declined for all CT scanners with lower tube current, PCCT showed a tendency towards superior image regarding soft tissue noise, SDCT for bone noise and DECT for diagnostic quality without reaching statistical significance (see Figure 3). Analysis of IRR showed substantial agreement (Fleiss’ κ of 0.68).

While the overall quantitative image quality was high for image datasets with maximum DLP, PCCT showed superior image quality in terms of SNR both at maximum DLP (SNR 10.3 vs. 4.4 at 100 mAs) and at minimum needed DLP compared to DECT (SNR 6.6 vs. 2.8 at 10/25 mAs, see Supplement S1).

At maximum settings (100 kV, 100 mAs for all scanners), DECT had the lowest overall DLP (8.1 mGy*cm) compared to PCCT (16.8 mGy*cm) and SDCT (111 mGy*cm). With respect to the minimum tube current at a set tube voltage of 100 kV for successful IGS setup and satisfying accuracy, the minimally needed DLP was significantly lower for PCCT (10 mAs, 1.7 mGy*cm) compared to DECT (40 mAs, 3.1 mGy*cm) and SDCT (30 mAs, 33 mGy*cm, see Supplement S1).

This results in a relative reduction in the radiation dose by 45.1% for PCCT compared to DECT and 94.8% compared to SDCT at minimum needed DLP, respectively.

## 4. Discussion

PCCT offers the potential for ultra-high-resolution imaging, with an in-plane spatial resolution of 0.11 mm and a slice thickness of 0.2 mm, making it a superior imaging technology for IGS-based FESS and ESBS procedures [18]. Ultra-high-resolution imaging may also provide additional insights into specific pathologies and anatomies, such as the cochlear microanatomy in the context of patient-specific cochlear implantation [25,26]. This study provides substantial experimental evidence that PCCT-based IGS for FESS enables accurate navigation at significantly reduced radiation doses while maintaining high image quality. Ultra-low-dose PCCT-based IGS accuracy was consistent with published real-world IGS accuracies, which are generally accepted to be below 2 mm [27].

Our results demonstrate that PCCT significantly improves image quality and intraoperative IGS accuracy compared with conventional CT imaging which is in line with previous publications demonstrating improved visualization of osseous structures and reduced metal artifacts [18,19,20,21,28]. The photon-counting technology used in PCCT allows for improved noise reduction and contrast-to-noise ratios, resulting in clearer and more detailed images [29]. This improved image quality facilitates a better visualization of small structures and fine details, supporting better surgical planning and decision-making [30]. The high spatial resolution and contrast of PCCT images allow a superior visualization of the intricate structures of the sinuses, enabling surgeons to accurately navigate and localize the surgical instruments. This is likely to improve surgical outcomes and reduce complications.

Additionally, the use of PCCT also results in lower radiation dose compared with conventional CT imaging. The reduced radiation exposures of PCCT and DECT in comparison to SDCT is a result of X-ray beam hardening due to the use of tin filters. SDCT does not enable this feature [31,32]. Although SDCT imaging offers a softer and subjectively more pleasant image appearance, these advantages are achieved through a different imaging kernel and are associated with an increased radiation dose. This is of great importance, as CT imaging is frequently repeated to enable intraoperative IGS when previous patient imaging is unsuitable, particularly in the high rate of revision surgery [33]. Because the median age of FESS patients is relatively low, the reduction in radiation dose may significantly decrease the risk of radiation-related complications [17]. Moreover, the use of PCCT in the head and neck region reduces artifacts caused by foreign bodies, such as dental implants, which frequently interfere with diagnostic imaging and IGS—particularly in the maxillary region [21]. Taken together, these advantages suggest that PCCT is likely to be of significant benefit for patients undergoing maxillofacial epithetic rehabilitation, who require repetitive imaging, navigated biopsies for their underlying disease, and placement of image-guided osseointegrated implants [34].

Consequently, low-dose PCCT protocols may be established for such imaging applications. Patients undergoing CT imaging for diagnostic purposes may still benefit from higher-dose protocols due to the associated improvement in bone and soft tissue contrast. However, CT scans acquired solely for IGS may be performed with the lowest tube current of 10 mAs without compromising IGS setup and accuracy.

Despite these promising findings, the following limitations should be addressed: the study was performed with a single cadaveric head only. Therefore, the study design does not allow for statistical evaluation of interindividual variability or reproducibility. Nevertheless, the use of a single cadaver in this early research phase was a deliberate methodological choice intended to assess the technical feasibility, radiation dose, and image quality of the procedure under realistic anatomical conditions. Translation to an in vivo human setting will therefore be important to verify the present findings. Nevertheless, the study design included multiple standardized measurements, three independent expert raters, and repeated evaluations across tube currents and scanner types, providing robust internal validity despite limited external generalizability. In addition, only one IGS system was used for evaluation, and effects of image quality variability may differ between systems and navigation technologies (i.e., electromagnetic versus optical IGS). Furthermore, the higher DLP of SDCT is primarily attributable to the lack of a tin filter, which is a technical manufacturer-dependent technical limitation rather than an inherent flaw of SDCT technology itself. Finally, PCCT system availability remains limited at present; although this may improve over time, it contributes to the current trend toward centralization in the medical sector.

In conclusion, our study demonstrates that PCCT is a valuable technology for imaging the paranasal sinuses. PCCT-based imaging improves the intraoperative accuracy of IGS, provides higher image quality, and reduces radiation dose compared to conventional CT imaging. Therefore, it may contribute to making fess—and especially the use of IGS–safer. Future studies should investigate the everyday application and long-term outcomes of PCCT use in FESS and ESBS to further establish its clinical utility.

## Data Availability

The datasets used and/or analyzed during the current study are available from the corresponding author on reasonable request.

## Supplementary Materials

The following supporting information can be downloaded at: https://www.mdpi.com/article/10.3390/diagnostics15212777/s1, Supplement S1: (**a**) Analysis of minimally needed dose length product for successful use of image-guided surgery (IGS). At a set tube voltage of 100 kV, minimal tube current for successful was significantly lower for photon-counting computed tomography (PCCT, 10 mAs, 1.7 mGy*cm) compared to dual-energy dual-source computed tomography (DECT, 40 mAs, 3.1 mGy*cm) and spectral detector computed tomography (SDCT, 30 mAs, 33 mGy*cm). (**b**) Analysis of objective image quality at minimally needed dose length product for successful use of IGS. PCCT showed superior image quality in terms of SNR both at maximum DLP (SNR 10.3 vs. 4.4 at 100 mAs) as well as at minimally needed DLP compared to DECT (SNR 5.3 vs. 2.8 at 10/25 mAs). SDCT imaging was only subjectively rated due to incomparability of the different manufacturers.

## Abbreviations

CTDI_vol	Computed Tomography Dose Index-Volume
CdTe	Cadmium Telluride
CNR	Contrast-to-Noise Ratio
CRS	Chronic Rhinosinusitis
CT	Computed Tomography
DECT	Dual-Energy Dual-Source Computed Tomography
DLP	Dose Length Product
EID	Energy-Integrating Detector
ESBS	Endoscopic Skull Base Surgery
FESS	Functional Endoscopic Sinus Surgery
IGS	Image-Guided Surgery
PCCT	Photon-Counting Computed Tomography
ROI	Region of Interest
SDCT	Spectral Detector Computed Tomography
SNR	Signal-to-Noise Ratio
VAS	Visual Analog Scale
