# Supplementary material for: Photon-Counting Computed Tomography of the Paranasal Sinuses Improves Intraoperative Accuracy of Image-Guided Surgery
Source: Diagnostics (Basel). 2025 Oct 31;15(21):2777. doi: 10.3390/diagnostics15212777 (PMC12608828; doi:10.3390/diagnostics15212777)
Supplement: Supplementary file 1 [file diagnostics-15-02777-s001.zip › diagnostics-3935586-supplementary.pdf]

Supplementary Material

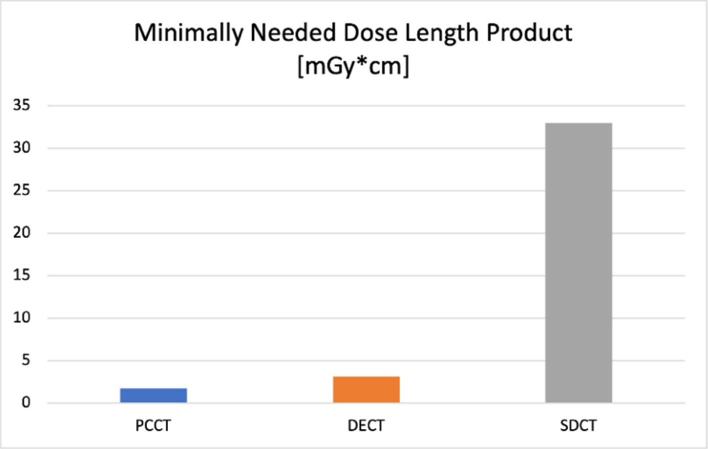

a

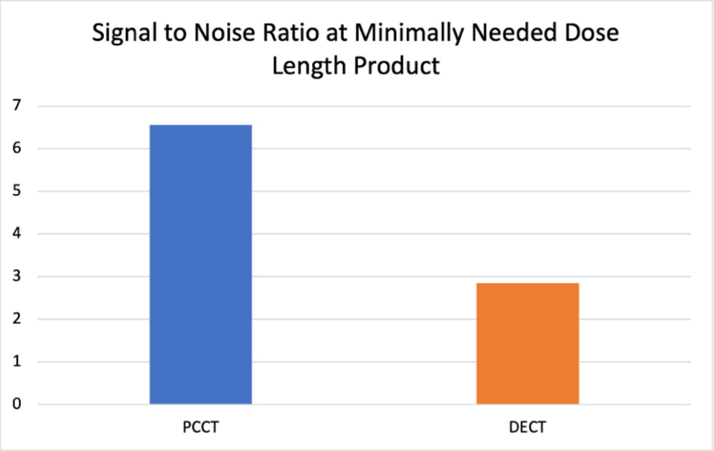

b

Supplement S1: a Analysis of minimally needed dose length product for successful use of image-guided surgery (IGS). At a set tube voltage of 100 kV, minimal tube current for successful was significantly lower for photon counting computed tomography (PCCT, 10 mAs, 1.7 mGy*cm) compared to dual energy computed tomography (DECT, 40 mAs, 3.1 mGy*cm) and spectral detector-based computed tomography (SDCT, 30 mAs, 33 mGy*cm). **b** Analysis of objective image quality at minimally needed dose length product for successful use of image-guided surgery. Photon counting computed tomography showed superior image quality in terms of SNR both at maximum DLP (SNR 10.3 vs. 4.4 at 100 mAs) as well as at minimally needed DLP compared to dual energy computed tomography (SNR 5.3 vs. 2.8 at 10/25 mAs). Spectral detector-based computed tomography imaging was only subjectively rated due to incomparability of the different manufacturers.
